# ‘You’ll Never Walk Alone’: A Qualitative Sequential Multimethod Study of Football‐Based Bereavement Support for Men Who Have Experienced Baby Loss

**DOI:** 10.1155/nrp/4511827

**Published:** 2026-07-30

**Authors:** Paula Carroll, Marie Kelleher, Mark Henderson, Andrew Hanlon, Bob McGurrell, Yana Richens, Jeanette Moore, Abigail Shevlin, Sean Coleman, Jane Rooney, Lucy Bray

**Affiliations:** ^1^ LFC Foundation, Liverpool Football Club, Liverpool, UK; ^2^ Faculty of Health, Social Care and Medicine, Edge Hill University, Ormskirk, UK, edgehill.ac.uk; ^3^ Honeysuckle FC, Liverpool Women’s Hospital, Liverpool, UK, nhs.uk; ^4^ 4Louis, Sunderland, UK; ^5^ Midwifery, University Hospitals Liverpool Group, Liverpool, UK

## Abstract

**Background:**

Despite men demonstrating high levels of grief and mental health difficulties after baby loss, support services predominantly focus on women. There is little known about the role of sport, football and targeted peer support for men who have experienced baby loss.

**Aim:**

To examine men’s experiences of accessing football‐based baby loss bereavement support and the self‐reported impact of the sessions on men’s grief and mental health. This project was designed with three men who have experienced baby loss.

**Methods:**

The study used a sequential QUAL–qual multimethod exploratory design. An online anonymous qualitative survey was distributed to all men who had attended a football session. A group discussion then considered the findings from the survey to add further depth to our understanding. Data were analysed using qualitative content analysis. Consent was obtained from all participants. Staff with mental health training were present during the discussion.

**Findings:**

Twenty‐one survey responses were received, and 11 men participated in the group discussion. Participants reported the limited targeted support available for men after baby loss, and that the football sessions provided a safe space to talk with other men who had a shared journey and experiences and that the football focus helped break down barriers and challenge the myths surrounding men and grief. The football‐based sessions were described as life‐changing and life‐saving.

**Discussion:**

The study highlights the need to dispel myths surrounding men’s grief after baby loss and emphasises the importance of football‐based bereavement support in addressing men’s mental health.

## 1. Introduction

The term baby loss is used as an umbrella term encompassing any loss of a pregnancy or baby, including miscarriage, stillbirth, and neonatal death [1]. Despite the growing recognition of the potential psychological and emotional impacts of pregnancy and baby loss on expectant parents, the majority of literature and subsequent care guidelines focus largely on women’s experiences [2]. This is despite research which highlights the high levels of grief experienced by men who have experienced pregnancy loss and neonatal death [3–5], with men shown to demonstrate significant symptoms of anxiety, depression and posttraumatic stress disorder [6]. Men have also reported that social taboos and gender stereotypes can marginalise their feelings and influence the provision of bereavement support [7]. Gender‐based grieving perceptions can lead men to conceal or internalise their emotions [8, 9], and evidence suggests that whilst intense grief reactions and posttraumatic psychological sequelae diminish over time in mothers, these often persist in fathers [10].

There is a notable and continued lack of support services available specifically for men who have experienced baby loss, both within the maternity and neonatal health care services and within community organisations [3, 11]. An independent pregnancy loss report from the Department of Health and Social Care in the United Kingdom (UK) (2023) [12] highlighted that current mental health provision is not sufficient to meet the needs of those who have experienced baby loss and stressed the importance of psychosocial support for both parents, recognising that men’s mental health, whilst integral to the family, is often overlooked. Seeking men’s perspectives of support for baby loss is important, given the noted gendered differences concerning help‐seeking, service access, grief styles [4, 6, 8] and peer support more generally. There is very little evidence regarding the development, delivery or impact of support for men’s baby loss grief, with limited research examining the effectiveness of interventions in this area [10, 13, 14]. There is also a call for more investment in support services for both parents and specific recommendations to challenge gendered stereotypes and support conditions for men to express related emotions [8]. Often, support services typically align with traditional therapeutic models such as talking therapy and are mainly aimed at women. Whilst there is emerging evidence demonstrating the positive impact football clubs have in engaging men in mental health promotion [15, 16] very little is known about the role of football‐based bereavement support for men who have experienced baby loss.

Honeysuckle FC is a collaboration between Liverpool Women’s Hospital and the LFC Foundation, Liverpool Football Club’s official charity; it is specifically for bereaved men who have experienced baby loss. The aim of the biweekly sessions is to provide a safe environment for the men. The sessions start with an hour of talking in a dedicated changing room space (called ‘changing room chats’). Here, the men discuss how their week has been through a supportive networking session, allowing people to talk, give advice or share coping strategies with the support of clinical staff, the LFC Foundation mental health team and their teammates. They then participate in an hour‐long football session followed by an optional social activity. This initiative began in November 2022, and although most of the group are local to the area, there are men travelling over 50 miles to be part of the team.

This research study aimed to examine men’s experiences of accessing Honeysuckle FC and the self‐reported impact of the service on their well‐being and grief. The study also aimed to gain insight into the perceived barriers to men accessing support after the loss of a baby and what would improve the bereavement support offered to men at an individual, family, community and policy level.

## 2. Methods

### 2.1. Study Design

This paper reports this study according to the Standards for Reporting Qualitative Research [17] (Supporting file 1).

The study, underpinned by a qualitative descriptive approach [18], used a sequential multimethod QUAL–qual exploratory design [19] to describe and interpret the experiences of men attending football‐based bereavement support. Qualitative data were collected using an online qualitative survey and a group discussion with men. The use of both methods aimed to make the investigation more comprehensive, making the research richer and more useful [19, 20] through an in‐depth understanding of the men’s experiences of attending the group whilst also providing them with the opportunity to share their views individually and anonymously.

### 2.2. Public Involvement

Public involvement was important in ensuring an acceptable and sensitive approach and is reported in this paper according to the GRIPP‐2 short form [21] (Table 1). A core group of three men who have experienced baby loss and also volunteer for Honeysuckle FC formed a Dad’s Advisory Group (DAG). These three men were consulted (via email, online meeting, phone call or face to face) on all aspects of the research design, including recruitment and data collection methods and materials, helping to shape the wording and questions of the online survey, the questions and format of the group discussion, including who should moderate it, and the format and content of the flyer and information sheets.

**TABLE 1:** GRIPP‐2 short form for reporting public and patient involvement [21].

Section	Overview
Aim	To ensure that the study addressed issues of importance to men attending Honeysuckle FC, that methods were inclusive and sensitive and that reporting of the findings was clear
Method	Three men were consulted via Teams, and through sharing iterations of the survey and group discussion questions, and developing analysis for feedback via email
Outcome	Consultations resulted in survey questions being amended to include more open‐ended questions. Consultation also informed the recruitment information (e.g., the design of the study flyers, the language used and the use of WhatsApp for recruitment
Discussion	Consultation was important in ensuring the survey addressed issues of importance to men attending Honeysuckle FC and was important when discussing interpretation during analysis
Reflection	The team ensured space and time to ensure consultation was conducted sensitively; this was facilitated by the men being supported in established parallel advisory roles

### 2.3. Participants and Recruitment

All men who had attended a Honeysuckle FC session were invited to take part. The convenience sample consisted of the men who volunteered to take part.

Men were made aware of the online survey by a flyer which was distributed by the Honeysuckle FC leads during the usual face‐to‐face sessions and posted on the established WhatsApp group. Using the WhatsApp group enabled those men who no longer attended Honeysuckle FC to have the opportunity to share their views. The flyer included a link to the online survey, which was open for 2 months in September–October 2024.

Recruitment for the group discussion was through a separate flyer distributed during the Honeysuckle FC sessions and posted on the WhatsApp group. The information about the group discussion was posted at least 2 weeks before the planned discussion to enable men time to read the information, ask questions and make a decision about participation. Men were prompted to contact the research lead if they wanted to take part or had any questions.

### 2.4. Data Collection

#### 2.4.1. Online Qualitative Survey

The short online qualitative survey was designed specifically for this research study, with input from the DAG who helped inform the questions. The use of surveys to collect qualitative data is increasingly recognised as a credible method [22] and was relevant in this study to explore the men’s experiences and perceptions of bereavement support. The online survey consisted of 14 open‐text questions, prompting men to share their experiences of attending the Honeysuckle FC sessions, the reported impact of attending the group on them and their family and asked them to identify any changes which would improve Honeysuckle FC (Supporting file 2). The survey was designed to take about 15–20 min to complete and was anonymous to enable men to honestly share their views and experiences; the men were reminded not to share any personal details within their responses. The survey was administered within Microsoft Forms, as this was a format familiar to the men and included an immersive reader option. The end of the survey included links to support services in case taking part prompted any men to become upset.

#### 2.4.2. Focus Group Discussion

The focus group discussion aimed to be a valuable addition to the survey data by asking the participants to reflect and collectively discuss [23] the provision of bereavement support through Honeysuckle FC. The focus group was held an hour before a usual Honeysuckle FC meeting. The group discussion involved the themes from the survey data being presented to the men and asking them to reflect on the findings and add further depth to our understanding. This format meant that men were not expected to share direct personal and sensitive experiences within the group discussion. Time was spent prior to the start of the group discussion outlining the key ‘ground rules’ of the group session, for example, respecting people’s opinions and not sharing information outside the group about others. The group discussions were facilitated by two female team members who were not linked to the delivery of Honeysuckle FC (LB and PC). The facilitators adopted a casual and conversational approach to the discussion. Large sheets of paper with the themes and some anonymised quotes from the survey data were positioned in the middle of the group, and men were prompted at points throughout the discussion to add their views either for a facilitator to scribe or to write them directly onto the sheets of paper or post‐its. These moments of movement and engagement helped reduce power dynamics and provide an informal atmosphere. It was made clear to the men that once they had taken part in the discussion, they would not be able to withdraw their data, as it would be too difficult to separate out an individual’s contribution. Additional staff with mental health training were present in an adjacent room in case any men became upset during the discussion. The group discussion was audio‐recorded.

### 2.5. Analysis

The data from the online survey were analysed using conventional qualitative content analysis techniques [24]. Two members of the team, who were female and experienced qualitative researchers (initials to be inserted after review), independently and inductively coded the open text qualitative responses from the survey and then met to discuss the codes. Any disagreements were discussed, and some codes were reshaped and labels changed. The codes were then further organised into themes. The themes were refined iteratively through discussion with the wider team. These themes developed from the survey data were taken to the focus group discussion; this led to further insights and clarification of interpretation.

### 2.6. Ethical Considerations

Ethical approval was gained from the author’s institution (ETH2223‐0336). The beginning of the online survey opened with the key information about participation and signposted men to the full information sheet stored online. Consent for survey participation was gained by the men confirming they had read the study information and by submitting the online survey. Men were aware that participation was voluntary and anonymous, and their responses would not be seen by the Honeysuckle FC facilitators. It was clear that once men had submitted the online survey, they could not withdraw their data due to participation being anonymous. The researchers gained written consent for all men taking part in the focus group discussion. It was clear to participants that once they had taken part in the group discussion, they could not withdraw their data, as they were part of a multiparticipant discussion.

### 2.7. Findings

#### 2.7.1. Participant Demographics

Twenty‐one men answered the online survey (68% response rate) and 10 men took part in a group discussion.

At the time of data collection, Honeysuckle FC had been running for almost a year. Most men (*n* = 11/21) who answered the survey had been attending Honeysuckle FC for more than 8 months. Most men reported in the survey that they had heard about Honeysuckle FC via the Liverpool Women’s Hospital Honeysuckle team or bereavement midwife (*n* = 16/21). A smaller number (*n* = 5/21) learned about Honeysuckle FC via other sources such as social media (Facebook), family members and other men.

The qualitative data from the surveys and group discussion are presented under the three themes: the lack of appropriate support for bereaved men, the men’s experience of Honeysuckle FC and the reported impact of the football‐based sessions. Quotes are anonymised and reported according to participant number and ‘S’ for survey respondents and just ‘FG’ for all focus group participants, as it was too difficult to determine individual voices on the recording.

#### 2.7.2. ‘There Was Nothing Out There for Me,’ a Lack of Appropriate Support for Men Experiencing Baby Loss

The men reported, in the survey and in the group discussion, how they had faced challenges in identifying or accessing appropriate support services for their grief before arriving at Honeysuckle FC. Men discussed that there was ‘*nothing out there*’ for them after they experienced baby loss and how they had experienced gender differences in the support offered. Men described how ‘*There is not much support out there for men*’ *(S-P5).*

Many of the men also discussed how support had been targeted towards their partner and that *‘…it is all designed for women, and what women go through, you know, I found nobody who gets it…and that’s why I didn’t speak to anyone for a year and a half, because I just couldn’t find any anyone’ (FG-P).*

*‘I didn’t feel like there was anybody who understood because it was all set up for dealing with women’ (FG -P).*

Men discussed at length how they felt the support they needed was different to their partner; the men who had been offered support in the form of talking therapies described how it had ‘felt awkward’ for them and ‘*it wasn’t for me*’ as ‘*I don’t really like talking, and I’m very good at kind of skirting around the topic if I’m not asked about it*’ (FG‐P).

The men also discussed the assumptions, myths and stereotypes that men perceive prevent them from accessing support. Many men discussed how they felt that society expects men to just ‘*get on with it*’ (FG‐P) and ‘*just carry on as normal*’ (FG‐P), and there are deep‐rooted beliefs that

*‘Men don’t grieve, men don’t cry. Having men being strong and the assumptions that they are fine’ (S-P11).*

Men also discussed that they felt that they had to ‘be strong’ for their partner and family, that they had to ‘*be the strong one and be there for everyone’ (S-*P9) and that needing support and expressing grief is perceived as a weakness.

One man discussed how these beliefs could lead to ‘*As a group of men, as men as a species, we get ignored a lot of the time, whether it’s in hospitals after that, after the event of a loss, just expected to go back to work and carry on as normal’ (FG-P).* This was despite mental health being discussed as a ‘*huge issue in men*’ (S‐P3) and that grief ‘*affects men as much as women*’ (FG‐P).

‘*The perception that you are not strong if you admit you’re hurting; it is in fact quite the opposite*’ *(S-P20).*

#### 2.7.3. ‘It is a Safe Space to Play Football and Share With People Who Understand’; Experiences of Football‐Based Bereavement Support

The men reported many positive experiences of attending Honeysuckle FC. The men discussed the safe space and trust fostered within Honeysuckle FC, which enabled them to share their feelings with men who had ‘*been through the same heartbreak*’ (S‐P15). The ability to open up and share experiences was facilitated by the group *‘only involving other men who have gone through the same thing and offers a safe space to talk’ (FG-P).* The men described not having to ‘*put on a brave face*’ (FG‐P).

This shared experience seemed to provide the men with the space to be ‘*authentically me*’ and openly acknowledge their grief without feeling judged, as the other men had ‘*been through baby loss and can offer the best advice*’ (S‐P15) and ‘*people don’t really understand it, if they’ve not been through it*’ (FG‐P). Many of the men’s accounts made it clear how important Honeysuckle FC was to them, going beyond a support group to forging ‘*Friends for life*’ and feeling ‘*like a family*’ (S‐P11).

The men discussed at length how football specifically helped them to develop relationships and break down any barriers to talking about their grief.

*‘The football acted as a buffer really and helped let my guard down to then talk to others before or after the sessions’ (S-P21).*

For some of the men, football was the reason they first attended Honeysuckle FC.

*‘When the baby passed, the bereavement midwife was saying come to this and I was saying no but as soon as the football was mentioned, I was like, that’s me*’ *(FG-P).*

Many of the men discussed how playing football helped them on many levels, with some describing how it helped them to ‘*blow off steam*’ (FG‐P), ‘*burn off some emotion*’ (FG‐P) and release what were often well‐guarded emotions, ‘*you’re already talking through football*’ (FG‐P)

#### 2.7.4. ‘It Has Helped Me Massively’; The Impact of Football‐Based Bereavement Support

The men reported in the survey and as part of the group discussion the multiple benefits and positive impacts they had experienced as a result of coming to Honeysuckle FC. The impacts were described as ‘*astronomical*’ (S‐P10), with men reporting how the support from Honeysuckle FC had improved their mental health and happiness.

*‘I honestly feel just a little bit happier in life even after this short time of going’ (S-P2).*

The men described how the sessions ‘*boosted my well-being massively*’ (FG‐P) and helped them *‘get back to being me’* (S‐P13). Some of the men reported the impact the support had had on their lives and well‐being as life‐changing.

*‘I truly believe I would be lost or 6ft under without being able to express my issues in a safe space that Honeysuckle FC provides*’ *(S-P3).*

Many of the men described how they had experienced isolation and reported that coming to Honeysuckle FC had helped them feel ‘*less alone in your grief*’ and more connected, with the sessions giving the men something to commit to and look forward to every week.

*‘It gets you off the couch. The more you sit there, the more you’re thinking and the more you’re going down and down and down’ (FG-P).*

The men also described how their confidence had increased as a result of coming to Honeysuckle FC, both in their ability to be open about their grief and in life more generally.

*‘It has helped massively with my confidence, I never thought I would have been able to walk into that room on that first day, so I feel like that has helped me in my day to day life too’ (FG-P).*

Many of the men discussed how the support from Honeysuckle FC had made a ‘*significant improvement*’ (FG‐P) in how they could talk about their grief with others and be more open with their partners and family.

*‘My home life has increased massively. Having the space to talk has allowed me to have the down times and let my wife be the support instead of the other way. I’ve always been open with our loss but now I have more confidence talking about it’ (FG-P).*

Attending Honeysuckle FC was described by the men as having ‘*helped me massively in my recovery, helped my partner and our families too’* (S‐P14). This also facilitated more openness between men and their partner and families; Honeysuckle FC also provided the men with a place to ‘*To say things that I can’t say to friends or even my wife because they wouldn’t understand*’ (S‐P3).

*‘Having somewhere where I can talk without worrying about upsetting or worrying my wife was perfect for me*’ *(S-P4).*

## 3. Discussion

We believe this is the first study to examine the reported impacts of football‐based support for men bereaved through baby loss. The study has identified the role of this football‐based support in providing a safe environment for men to explore their emotions, share experiences and receive invaluable peer support from people who had also experienced grief from baby loss. The study also highlights men’s unique support needs and the persistent gendered societal stigmas which can lead men’s grief to be overlooked by traditional support systems.

The findings from this study demonstrate the huge impact baby loss can have on men’s mental health, supporting previous evidence which shows that self‐reported depression can last 3–5 years following the loss of a child [25], and that in the first 6 months following the death of a child, fathers experience an increase in acute illnesses, hospitalisations and medication change, which can be persistent for many years [26]. These challenging experiences exist against a backdrop of noted gendered differences, as many of the men described putting their own grief to the side to support their partner and family. This resonates with how fathers in other research have also been shown to deflect attention from their suffering to prioritise support for their partners [27]. Aydin and Kabukcuoğlu [3] coined the term ‘neglected group of fathers’ in their meta‐synthesis and discovered how patriarchy and men feeling the need to be strong and a protector of the family have a significant impact on men’s grief.

This study also highlighted that bereavement and support services following baby loss were not accessed by the men, compared to their partners. Furthermore, the men were offered services which were not perceived to meet their needs. This supports previous evidence demonstrating that men are less likely to access psychological therapies than women [28], tend to grieve more in isolation than in public [5, 10] and avoid discussing their feelings of grief in contrast to more open communication by mothers [29–34]. It is recognised that less formal and different approaches to support might help men struggling with grief but who are not keen to engage in traditional talking therapies [35] and that men would be more likely to seek help if therapy or support aligned more with men’s preferences [36].

The men in this study described how the football sessions provided this ‘different model of bereavement support’ to talking therapies, addressing their needs in multiple ways. Football gave the men a physical outlet for their emotions and fostered a safe space to share feelings with other men who had ‘walked in their shoes.’ The football sessions reduced men’s ‘isolation in grief’ and the sport activity, which is not centred on emotions, allowed the men to create positive trusted relationships. The sessions tackled the ‘suffering in silence’ many of the men described and is evidenced in previous research [14, 29–34].

The positive reported impact of the football‐based bereavement support aligns with research which suggests that involvement in sports has positive influences on men’s grief outcomes and mental health [37] and adds to the understanding of the role of football‐based interventions in improving men with mental health difficulties [38, 39].

The football‐based bereavement sessions were able to break down some of the myths described by the men. This study highlights the need for dedicated spaces such as Honeysuckle FC to support men’s grief following baby loss. There are opportunities to replicate the model across different geographical areas. This study has provided valuable insight into how Honeysuckle FC provides men with a supportive space for their baby loss grief and has provided a platform for further research into the role of football and sport in facilitating bereavement support for men (Table 2).

**TABLE 2:** Contribution of the study to the existing literature.

Issue	Despite men demonstrating high levels of grief and long‐term mental health difficulties after baby loss, support services typically align with traditional therapeutic models such as talking therapy and are mainly aimed at women
What is already known?	Sport, football and targeted peer support can impact positively on men’s mental health
What this paper adds?	Football‐based bereavement support has an important role in addressing men’s grief and mental health after baby loss

## 4. Strengths and Limitations

While interesting, useful and in‐depth information was collected, this was a small study which involved a self‐selecting sample. Even though over two‐thirds of the group shared their views, it is unknown whether those with more negative views of Honeysuckle FC chose not to participate. A further limitation is that the study was conducted in England, where football is the most popular sport and plays an important role in many people’s lives; this may limit the transferability of the findings to other countries.

The main strength of the study was the design, conduct, interpretation and dissemination being informed and shaped by three men with lived experience of baby loss; this helped the team research in a sensitive manner.

## 5. Conclusions

The study shows that the Honeysuckle FC football‐based bereavement sessions fill a notable gap in the provision of support to men who have experienced baby loss. The combination of football and facilitated discussion to enable the men to share feelings with those who have experienced similar life events within a safe space made Honeysuckle FC ‘life‐saving’ and ‘life‐changing.’

## Funding

No funding was received for this research.

## Conflicts of Interest

Three of the authors (Paula Carroll, Mark Henderson and Andrew Hanlon) are employees of Liverpool FC Foundation, and Lucy Bray is part of the LFCF advisory board. The authors believe that this employment or voluntary relationship does not constitute a conflict of interest. While these authors are affiliated with the organisation that funds the programme, all authors have endeavoured to ensure that the research was conducted and reported in a transparent way, and we remained open to hearing negative as well as positive outcomes.

All elements of the development and conduct of the project were overseen by the wider team, of which many had no affiliation to LFCF. The remaining authors declare no conflicts of interest.

## Supporting Information

Additional supporting information can be found online in the Supporting Information section.

## Supporting information


**Supporting Information 1** Supporting File 1: Standards for Reporting Qualitative Research (SRQR).

**Supporting Information 2** Supporting File 2: Questionnaire used in the study.

## Data Availability

The data that support the findings of this study are available on request from the corresponding author. The data are not publicly available due to privacy or ethical restrictions.
