# Supplementary material for: ‘You’ll Never Walk Alone’: A Qualitative Sequential Multimethod Study of Football‐Based Bereavement Support for Men Who Have Experienced Baby Loss
Source: Nurs Res Pract. 2026 Jul 30;2026:4511827. doi: 10.1155/nrp/4511827 (PMC13420737; doi:10.1155/nrp/4511827)

Honeysuckle FC Questionnaire

Honeysuckle FC

Please read this important information before completing this survey.

Honeysuckle FC is a collaboration between the Liverpool Women's Hospital and the LFC Foundation. It is a football team that brings together bereaved men who have been affected by baby loss.

We are inviting you to share your views about Honeysuckle FC to help us work out what it is like to come along to the sessions, what works well and if there is anything we need to change.

This project is being carried out by people from the LFC Foundation, Liverpool Women's Hospital Honeysuckle Team and Edge Hill University. Please make sure you have read the full information sheet about this project before you start the survey (link).

This survey is for any Dad who has been to a Honeysuckle FC session.

We think the survey should take about 5-10 minutes to complete, but may take a bit longer if you have lots you want to tell us.

The survey is anonymous - no-one running the Honeysuckle FC sessions will know your answers.

If you click 'done' at the end of the survey then this means you are happy for us to use your answers as part of the project. As it is anonymous, once you have clicked done or submit then you cannot take your answers back.

There is a button to click at the beginning of the survey to confirm you have read the information about the study and are happy to take part. At the end of the survey there are some suggestions for places for support, you can skip straight to these if you like. As we cannot identify who has filled out a survey, we will not be able to offer individual help or support.

If you have any questions or concerns please contact Paula on paula.carroll@liverpoolfc.com or Lucy on brayl@Edgehill.ac.uk

Thank you!

1. How long have you been coming along to the Honeysuckle FC sessions?

Enter your answer

2. Tell us what led you to come along to the Honeysuckle FC sessions?

You can tell us as little or as much as you like here. Please also feel free to skip this question.

Enter your answer

3. How did you hear about the Honeysuckle FC?

Enter your answer

4. Have you been offered, or have you accessed, any other bereavement services or support? If so what were you offered and did you access the service?

Enter your answer

5. What is it about Honeysuckle FC that appealed to you over other men's support services?

Enter your answer

6. What impact or change has coming along to Honeysuckle FC had on your wellbeing?

Enter your answer

7. What impact or change has coming along to Honeysuckle FC had on your relationship with friends and family?

Enter your answer

8. What impact or change has coming along to Honeysuckle FC had on your day to day life including work and socialising?

Enter your answer

9. What have you found most helpful or liked most about Honeysuckle FC?

Enter your answer

10. Is there anything you dislike about Honeysuckle FC?

Enter your answer

11. What could be done differently to improve Honeysuckle FC?

Enter your answer

12. What assumptions/ perceptions / myths / stereotypes do you think need challenging about men experiencing baby loss/grief?

Enter your answer

13. If you no longer come along to the sessions can you tell us why?

Enter your answer

14. Is there anything else that you want us to know?

Enter your answer

Submit

This content is created by the owner of the form. The data you submit will be sent to the form owner. Microsoft is not responsible for the privacy or security practices of its customers, including those of this form owner. Never give out your password.

Powered by Microsoft Forms |
The owner of this form has not provided a privacy statement as to how they will use your response data. Do not provide personal or sensitive information.
| Terms of use

Questionnaire as displayed on a mobile

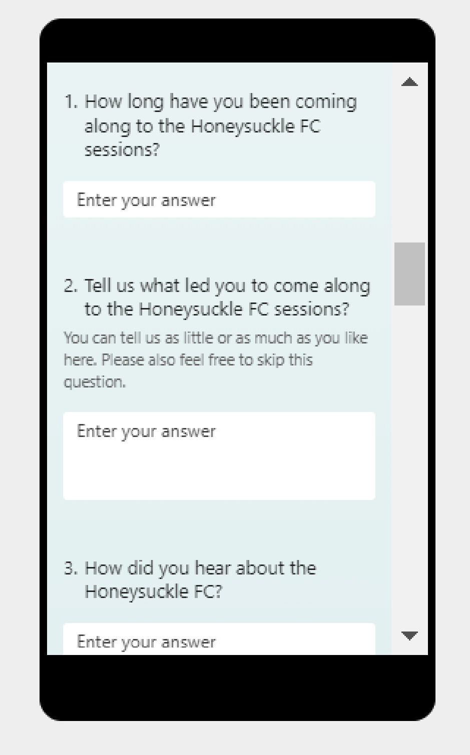

Supplement: Supplementary file 2 — Supporting Information 2 Supporting File 2: Questionnaire used in the study. [file NRP-2026-4511827-s001.pdf]
